# Commentary: Development and immunogenicity evaluation of a quadruple-gene-deleted pseudorabies virus strain

**DOI:** 10.3389/fmicb.2025.1665381

**Published:** 2025-10-13

**Authors:** Yan Li, Chunlin Mu, Feifei Yin, Fuxiao Liu

**Affiliations:** ^1^College of Veterinary Medicine, Qingdao Agricultural University, Qingdao, China; ^2^Qingdao Center for Animal Disease Control & Prevention, Qingdao, China

**Keywords:** pseudorabies virus, quadruple-gene-deleted mutant, UL24, vaccine, immunization

## Introduction

An article, named *Development and immunogenicity evaluation of a quadruple-gene-deleted pseudorabies virus strain* (doi: 10.3389/fmicb.2024.1479794), was recently published in *Frontiers in Microbiology* (Li et al., 2024). In this study, Li et al. used the CRISPR/Cas9 technique for modifying a pseudorabies virus (PRV) strain to generate a mutant, albeit deficient in four genes, showing similar growth kinetics to that of its progenitor. After vaccination with the quadruple gene-deficient mutant, neither mice nor piglets displayed obvious clinical signs and pathological alterations. The mutant induced significantly higher levels of gB-specific antibodies, neutralizing antibodies and cytokines than both the Bartha-K61 strain and a triple gene-deficient mutant did. Moreover, compared with other vaccine strains, the quadruple gene-deficient mutant conferred robust protection against challenges with a virulent PRV in mice and piglets. Herein, we would like to express our scientific opinions on this study.

## Characteristics of PRV

**Figure 1:**
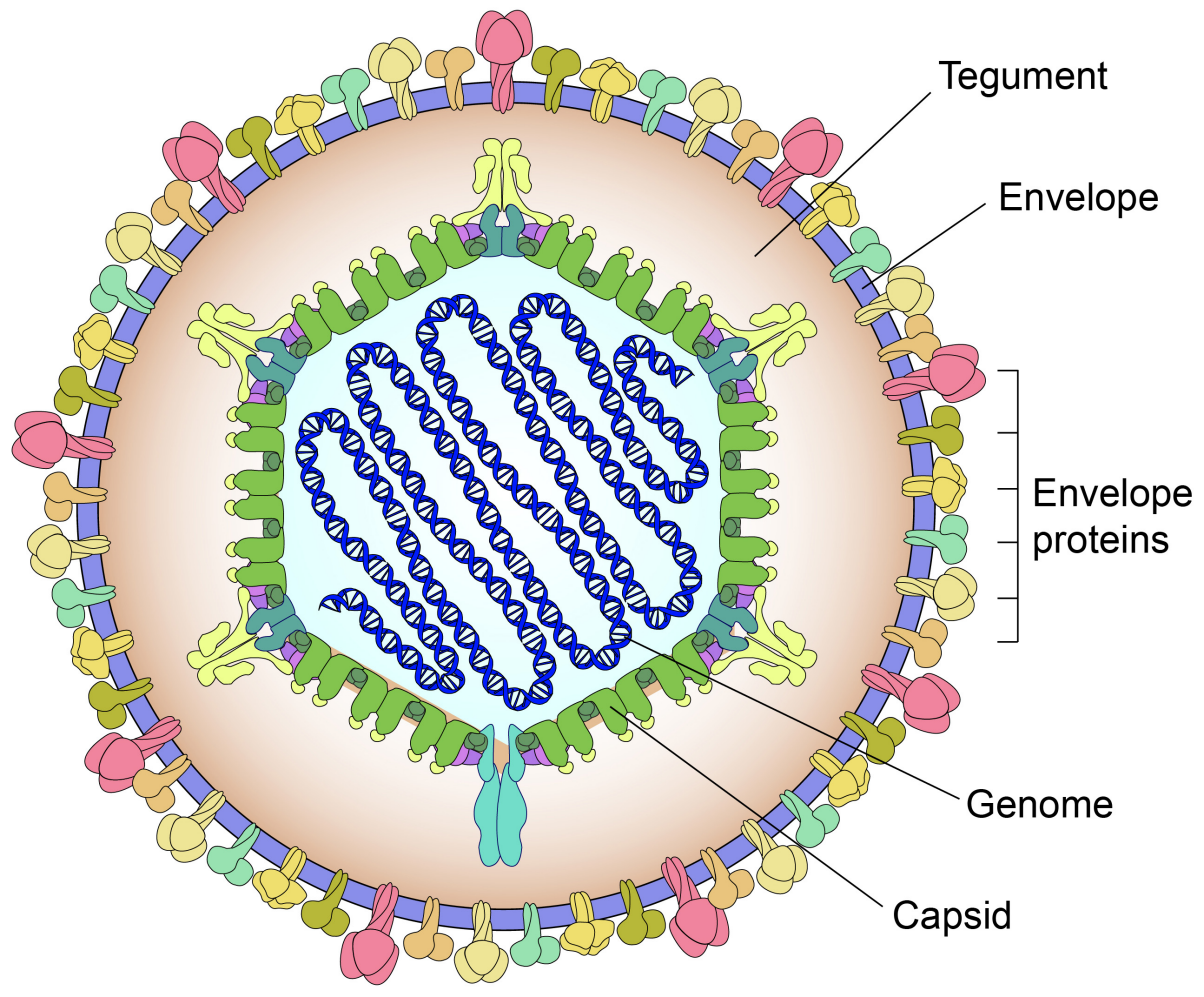
Schematic representation of pseudorabies virion. The virion is composed of four morphologically distinct components: a linear double-stranded DNA genome, an icosahedral capsid, an amorphous tegument layer, and a lipid envelope studded with various glycoproteins.

PRV causes Aujeszky's disease that is particularly devastating to breeding sows and piglets. This virus belongs to the genus *Varicellovirus* in the family *Orthoherpesviridae*. An intact PRV virion is composed of four morphologically distinct components (Figure 1): a linear double-stranded DNA genome, an icosahedral capsid, an amorphous tegument layer, and a lipid envelope studded with various glycoproteins, out of which gE, gL, gG, gC, gM, and gN are the main virulence determinants (Peng et al., 2023). The genome includes a unique long region, a unique short region, an internal repeat sequence, and a terminal repeat sequence (Li et al., 2021). Its full-length sequence is approximately 143 kb, comprising at least 72 open reading frames that encode 70–100 proteins (Mettenleiter, 2000; Klupp et al., 2004).

## Development of gene-deleted vaccines against PRV

Conventional live-attenuated vaccines often fail to provide satisfactory effect on controlling PRV variants in recent years (Li et al., 2025). Gene-deleted strains have been extensively explored for the development of anti-PRV vaccine candidates (Zheng et al., 2022). Gene-deleted targets primarily focus on TK, gE, and gI genes, which play essential roles in PRV's neurovirulence, transmission or replication (Moormann et al., 1990; Wu et al., 2016; Sun Y. et al., 2022). Gene deletion alone or combinedly would attenuate the virulence of wild-type strains, facilitating the development of live-attenuated, gene-modified vaccines (Wang et al., 2014). A representative gene-modified strain is the SA215 (ΔgE/ΔgI/ΔTK), licensed in 2003 for producing commercially available vaccines. Other genes, such as gD, gG, US9, and US2, can be also deleted from the PRV genome for constructing vaccine candidates (Mettenleiter et al., 1994; Sun L. et al., 2022).

## Potential of quadruple gene-deficient mutant in immunization

PRV UL24 is a nuclear-localized protein. Compared with TK, UL24 functions as a minor virulence-associated factor (Ye et al., 2019), not only antagonizing the antiviral effect mediated by oligoadenylate synthetases-like protein (Chen et al., 2021), but also abrogating the NF-κB activation induced by tumor necrosis factor-α (Wang et al., 2020). Different from the classical vaccine strain SA215, the quadruple gene-deficient mutant, derived from another progenitor SX-10, was constructed by (Li et al. 2024) through deleting one extra UL24 gene.

The authors compared growth curves among different strains, showing that the quadruple gene-deficient mutant was insignificantly affected concerning its growth kinetics. Deletion of UL24 enhanced the level of interferon-β expression, and moreover reduced the PRV virulence in mice. Additionally, the ΔgI/ΔgE/ΔTK/ΔUL24 mutant-vaccinated group exhibited the antibody response significantly stronger than that of the ΔgI/ΔgE/ΔTK mutant-vaccinated group. Both groups revealed the similar titer of neutralizing antibodies at 28 days post-immunization, significantly higher than that of the Bartha-K61-vaccinated group. Further, (Li et al. 2024) evaluated the quadruple gene-deficient mutant regarding its safety and immunogenicity in piglets. The results showed that this mutant caused neither clinical signs nor histopathologic changes, and conferred the optimal protection from the challenge with virulent PRV (Li et al., 2024).

## Discussion

Pseudorabies has still been endemic in most parts of the world (Chen et al., 2025; Zhuang et al., 2025). Both gene-modified and -unmodified vaccines are concurrently used in the veterinary field for preventing pseudorabies, whereas the former possesses a better safety profile than the latter. It was reported as early as 1985 that TK-negative PRV was used to develop the gene-deleted vaccine for clinical immunization (Kit et al., 1985). A series of dual gene-deleted strains, such as ΔTK/ΔgI, ΔTK/ΔgE, ΔgD/ΔgI, and ΔgE/ΔgI, were subsequently constructed and also demonstrated to be highly immunogenic and safe in pigs against pseudorabies (Moormann et al., 1990; Peeters et al., 1994; Gu et al., 2015; Wu et al., 2016). Meanwhiles, triple gene-deleted PRVs have been continuously reported to be successfully developed, and also induced strong immune responses *in vivo* (Ferrari et al., 2000; Zhu et al., 2011; Hu R. M. et al., 2015; Lin et al., 2020).

TK, gE and gI were the three most frequently modified targets. In the study conducted by Li et al., besides the three genes, one extra virulence-related gene, UL24, was deleted from the PRV genome. Because the PRV UL24 functions as an innate immune antagonist in the host (Ye et al., 2019; Wang et al., 2020; Chen et al., 2021), its deletion would theoretically enhance the innate immunity. Indeed, regardless of the PRV UL24 deleted alone or in combination with gI, gE, and TK, the interferon-β transcription always remained at a relatively high level *in vivo* after the vaccination with UL24-deficient strains. The quadruple gene-deficient mutant could replicate as efficiently as wild-type strains *in vitro*, and more importantly, was able to elicit more robust immune responses than the triple gene-deficient (ΔTK/ΔgE/ΔgI) strain to some extent (Li et al., 2024). The quadruple gene-deficient mutant was derived from an emerging PRV variant, SX-10. Choosing a variant, rather than a classical strain, is necessary for developing the next-generation vaccine, because classical strains, albeit safe to use, may fail to confer the desirable immune protection (An et al., 2013; Hu D. et al., 2015).

In conclusion, this study is novel, to which similar findings were previously unreported, therefore paving the way for developing new-generation vaccines against pseudorabies in future. Further experiments should be designed for evaluating its immunization efficacy in field conditions.
